# Three-Dimensional Changes of the Auditory Canal in a Three-Year Period during Adolescence Using CBCTs

**DOI:** 10.1155/2018/5463753

**Published:** 2018-10-22

**Authors:** Adam Woods, Manuel O. Lagravère

**Affiliations:** ^1^Undergraduate Student, School of Dentistry, Faculty of Medicine & Dentistry, University of Alberta, ECHA, 11405-87 Avenue, Edmonton, Alberta, Canada T6G 1C9; ^2^Associate Professor, School of Dentistry, Faculty of Medicine & Dentistry, University of Alberta, Edmonton, Alberta, Canada

## Abstract

**Purpose:**

There is a lack of identifying suitable regions in the head that can be used for three-dimensional superimposition techniques. For this reason, with the use of cone-beam computed tomography (CBCT), the ear canals were analyzed to verify changes during a period of three years in the adolescent years.

**Methods:**

CBCTs from fifty-six patients (ages: 10 to 20) were used to landmark the anatomy of the ear canals. Each patient was analyzed using two CBCT reconstructions that were taken approximately three years apart. AVIZO® software was used to locate 28 landmarks distributed following the ear canal path and foramina (ovale, spinosum, rotundum, etc.) in the cranial base to obtain spatial relationships. Three-dimensional coordinates were obtained from the landmarks, and the average distance between various landmark pairings was calculated. The repeated measure ANCOVA was used to determine statistical significance.

**Results:**

In the main data set, the largest mean distance change was found to be 4.37 mm ±  18.29 mm between the left foramen ovale and the left superior medial ear canal opening. The smallest mean distance change was 0.18 mm ± 3.25 mm between the right inferior lateral ear canal opening and the right inferior medial ear canal opening.

**Conclusions:**

During the adolescent years, the ear canal presents dimensional changes. Even though in different areas throughout the canal, the average distances were minor, still, large standard deviations were present; thus, caution should be taken when trying to use this structure for superimposition of CBCTs.

## 1. Introduction

Cone-beam computed tomography (CBCT) has become a popular imaging tool for dental practitioners in North America to visualize structures in the head and neck in three dimensions (3D). While CBCTs struggle with the differentiation of very similar density soft-tissue structures, they produce a high-resolution image when compared to 2D imaging at a lower cost and lower radiation dose when compared to medical CT [1]. The 3D capabilities of CBCTs allow clinicians to better understand a patient's dental development, the potential for dental movement, and possible airway obstruction.

CBCTs have become useful in orthodontic treatment, allowing practitioners to monitor treatment efficacy [2]. Of particular interest are the advantages that CBCTs offer practitioners when trying to superimpose facial structures to assess treatment outcomes. Historically, superimposition has been done using two-dimensional (2D) lateral cephalograms, primarily focusing on relatively stable structures, including the sella turcica, lingual curvature of the palate, and inner border of the symphysis [3]. Evidently, the potential for cephalometric analysis and diagnosis is promising as we move towards the 3D capabilities of CBCT [4]. The caveat of new technology, of course, is that a lot of analysis and optimization is required to establish reliability. Various authors have developed methods of reducing error associated with superimposition of structures using CBCT, most typically using landmarks such as the left and right auditory meatus, the left and right infraorbitale, the left and right menton, the dorsum foramen magnum [5], anterior wall of the sella turcica, planum sphenoidale, and superior portion of the ethmoid bone [2]. In order to more effectively establish the efficacy of these structures as reference points, it is logical that they should be analyzed using CBCT to determine the extent to which they are changing over time.

The auditory meatus has been landmarked and measured through a number of methods including histological techniques [6], moulding [7], computed tomography imaging [8, 9], dissection [10], magnetic resonance imaging [9], and direct fluid measurements [11]. Previous investigation has indicated that while the auditory meatus undergoes a great deal of development during the embryonic, fetal, and childhood periods, development typically slows and finalizes during the adolescent years [7]. While the auditory meatus has been imaged 2D for use as a reference point in orthodontic treatment [12], it has not been investigated thoroughly in 3D.

A further understanding of the developmental processes that occur in the auditory meatus would have a positive outcome in orthodontic treatment planning. If this structure remains relatively stable in terms of dimensional changes throughout adolescent development, it is possible that it could be used as a reference structure. In the proposed study, images taken using CBCT will be used to landmark and assess development of the auditory meatus in adolescents over two time points. It is hypothesized that the auditory canal will present no significant change over these time periods.

## 2. Materials and Methods

**Figure 1:**
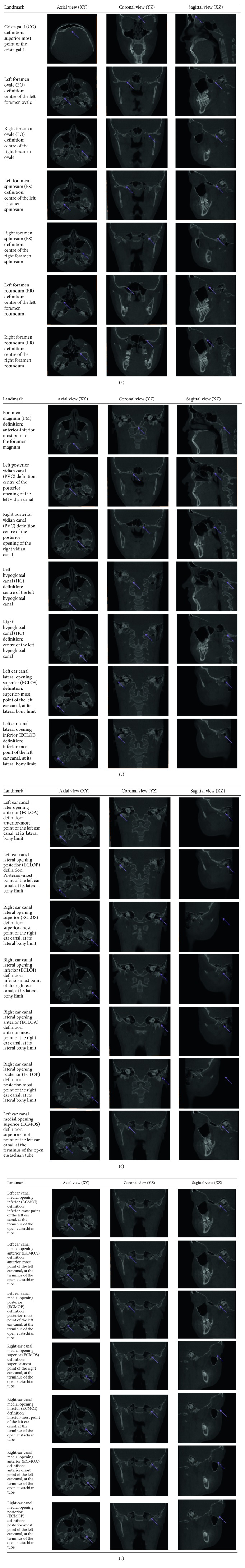
Landmarks used for reliability and distance analysis.

CBCTs of 56 patients were selected from the Graduate Orthodontic Clinic patient pool in Edmonton, Alberta. These patients were part of clinical trials involving analysis of CBCTs, and patients were informed that taking of CBCT is not the standard of care. With this, it is not suggested that CBCTs should be taken on everybody. Inclusion criteria for the patients were 10–20 years of age, full permanent dentition (with exception of third molars), and absence of syndromic characteristics or systematic disease. A power sample calculation was done considering 80% power and a 5% alpha level using data from Lagravere et al. [13] demonstrated that a sample of 25 patients per group was sufficient using a difference of 1.5 mm. Since we had more available, it was decided to analyze all the samples available in the database. Approximately three years existed between each patient's serial CBCTs, signalling the start and end of orthodontic treatment. CBCT scans were taken using the ICat New Generation (Imaging Sciences International, Hatfield, PA) machine at 0.3 mm voxel size and 8.9 seconds, 13 cm × 16 cm FOV, 120 kV, and 5 mAs with 8 mm aluminum filtration and converted into the DICOM format. The images were analyzed using Avizo 8.1 software (Visualization Science Group, New England, MA). Eight landmarks within each auditory meatus were located, including the superior-, inferior-, anterior-, and posterior-most aspects of the bony limit of the right and left ear canal laterally, and the termination of the Eustachian tubes medially (Figure 1).

As one observer performed the landmarking in this study, it was important to verify that the placement of each landmark could be replicated reliably. To do so, landmarks throughout the cranial base were located on 10 different CBCTs, each coming from a separate patient. From the ear canal landmarks, other 12 landmarks located in different structures of the cranial base were located. The selection of these extra 12 landmarks was based on analysis of the cranial base and selecting structures that can be easily located and recognized in the cranial base. This was repeated once a week for three weeks. In total, 28 landmarks were assessed in each patient, as shown in Figure 1 and Table 1. The three iterations of each landmark were then analyzed for consistency using a 95% confidence interval (CI 95). This was done by subtracting the coordinates of one trial with the ones of another trial (for example, Trial 1-Trial 2, Trial 1-Trial 3, and Trial 2-Trial 3), and then, these differences were averaged to obtain an average measurement error.

Once reliability was determined, CBCTs from the main patient pool were analyzed. The auditory canal was isolated and landmarked in each image so that various dimensions could be analyzed. After landmarks are located, distances were calculated using the following equation (Table 2):

(1) $$\begin{array}{c} d=\sqrt{{\left(X_{1}-X_{2}\right)}^{2}+{\left(Y_{1}-Y_{2}\right)}^{2}+{\left(Z_{1}-Z_{2}\right)}^{2}}. \end{array}$$

Analysis included serial patient images taken from two different time points, so that the change in dimension over time could be assessed. Descriptive statistics were calculated for all distances and for the differences between corresponding distances at the two time points. Sex and age distribution are shown in Table 3. A repeated measures analysis of covariance (ANCOVA) was applied to the data having age at baseline and gender as covariates. This analysis was done to verify if there was a statistical significant difference between both time points (*p*<0.05).

## 3. Results

In order to ensure reliability of the landmarks chosen, the CBCTs of ten patients were landmarked three times, with one week in between each landmarking session. The largest measurement error was found in the *Z*-coordinates of the left ear canal lateral opening posterior landmark (Figure 1) at 2.0 mm. The smallest measurement error was found in the *Z*-coordinates of the crista galli landmark at 0.1 mm. Intraclass correlation coefficient (ICC) is a commonly used tool to assess intraobserver reliability. In this study, the lowest ICC value was from the left anterior lateral ear canal landmark at 0.99 (CI 0.95–1.00). All landmarks were determined to have appropriate reliability (Table 1).

**Figure 2:**
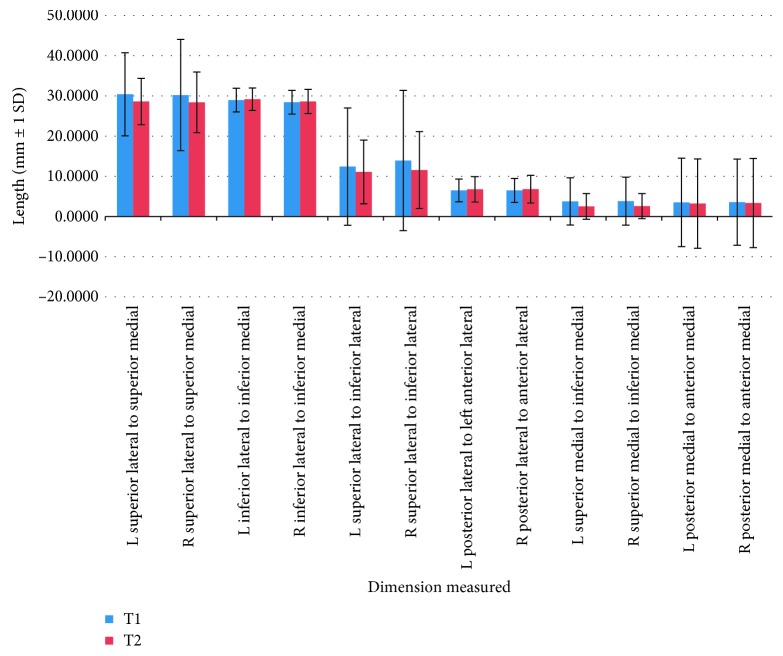
Graphic representation of the major distances in T1 and T2.

**Figure 3:**
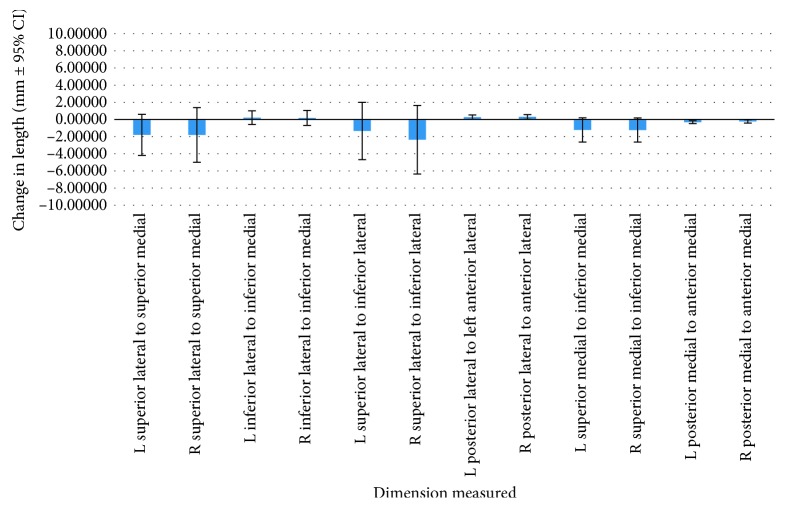
Graphic representation of the change in distances between T1 and T2.

Twenty-eight distances between 15 different landmarks were used to determine whether the ear canal changed significantly in vertical, anterior-posterior, and transverse dimensions throughout adolescent development. All distances analyzed did not present with a statistically significant (*p*>0.05) change over the time period when viewing the repeated measures ANCOVA test. When analyzing the raw distance changes (Table 2), it is observed that 6 of the 28 distances presented changes larger than 2 mm. These distances were mostly related to foramen ovale and foramen rotundum relationship. The rest of distances within the ear canal presented differences less than 2 mm. The greatest overall change was found to be 4.4 mm (Table 2), from the left foramen ovale to the left ear canal medial opening superior (Figures 2 and 3).

## 4. Discussion

The popularity of CBCTs in orthodontics has increased in recent years as a result of its enhanced 3D diagnostic information when compared to conventional radiographic imaging and its reduced radiation dose when compared to multislice CT [14, 15]. It should be noted that even if the radiation of CBCT is less than medical CT, it is still higher compared to traditional two-dimensional radiographs; thus, caution should be taken when assigning a patient to have a CBCT taken. In order to effectively monitor the effectiveness of orthodontic treatment, it is important to be able to reference orthodontic changes in relation to structures that remain stable over the treatment period. A number of studies have discussed the difficulty in establishing the reliability of landmarking the external auditory meatus [13, 16, 17]. In this study, landmarks were placed on the superior-, inferior-, anterior-, and posterior-most positions of the external auditory meatus just lateral to the point where it is surrounded in bone. We were unable to locate a study referencing a reliable landmark to represent the medial end of the ear canal. Additionally, due to the difficulty associated with identifying bony structures within the inner ear, landmarks representing the medial portion of the ear canal were placed in a reliably reproducible location at the end of the Eustachian tubes.

The majority of distances analyzed involving the ear canal did not show a significant change over the observed development period. Four of the five significant changes occurred in distances representing the heights and widths of the ear canal end points. This is most likely a result of differences in CBCT quality. These landmarks were placed on soft-tissue borders, even though they were referenced using neighbouring bony structures. As a result, in certain data sets, a difference in image quality could have influenced the placement of these landmarks to some degree. In most cases, the change seen in these distances was just over 1 mm, and as a result would likely be insignificant clinically.

A systematic review done by Lisboa et al. demonstrated that landmarking the porion presented with a large degree of interevaluator error [18]. Ludlow et al. specifically noted that the porion is difficult to landmark as a result of structure curvature, proximity to the temporal bone, and whether bony or soft-tissue contours were used [10]. While this study defined ear canal landmarks on soft-tissue borders, it is possible that the other factors influenced the variability seen in some of the reliability as well as the distance measurements. This may have caused the large standard deviations found in some of the measurements analyzed. Although average values were low, the large standard deviations showed that in some cases the dimensions reduced. Being this a new area in terms of three-dimensional analysis of the ear canal could not find other references with these results for comparison. It is expected that structures increase in size or become more separate to other structures, but in this case, we suggest to take the changes with caution.

### 4.1. Limitations

One limitation of this study involves the difficulty of which certain landmarks were defined. Specifically, the medial end of each ear canal was difficult to define due to some variability in CBCT quality, especially within the inner ear. As a result, a landmark was chosen within the Eustachian tube that was simpler to identify consistently. That being said, the different qualities likely resulted in some additional variance in the lateral and medial ear canal landmarks.

Another limitation involves the inability to use certain landmarks for every data set. In particular, the crista galli was not present in 8 of 12 reliability trials, as those CBCTs did not extend superiorly sufficiently enough to capture the landmark.

Another limitation was that the size of the ear canal was determined using only the distance between two 3D end points. A more thorough analysis could examine the volumetric change of the ear canal by providing landmarks from lateral to medial end points.

Finally, although the sample size indicated a correct power, more data should be collected in order to completely verify where the changes of the ear canal happen; thus, this should be considered a preliminary analysis.

## 5. Conclusion

In general, our results suggest that the ear canal presents changes throughout in terms of dimension and relationship to other structures in the cranial base. Even though in different areas throughout the canal, the average distances were minor, still, large standard deviations were present; thus, caution should be taken when trying to use this structure for superimposition of CBCTs.

## Figures and Tables

**Table 1:** Mean measurement errors in *X*, *Y*, and *Z* coordinates for each landmark.

Landmark	Mean			SD		
	X	Y	Z	X	Y	Z
Crista galli (CG)	0.16	0.47	0.10	0.07	0.05	0.12
Left foramen ovale (FO)	0.51	0.52	0.55	0.33	0.31	0.50
Right foramen ovale (FO)	0.55	0.45	0.47	0.36	0.25	0.27
Left foramen spinosum (FS)	0.28	0.31	0.45	0.18	0.20	0.22
Right foramen spinosum (FS)	0.30	0.34	0.48	0.22	0.11	0.28
Left foramen rotundum (FR)	0.35	0.62	0.23	0.24	0.44	0.08
Right foramen rotundum (FR)	0.48	0.79	0.41	0.31	0.91	0.48
Foramen magnum (FM)	0.41	0.43	0.31	0.21	0.17	0.13
Left posterior vidian canal (PVC)	1.04	1.15	0.50	1.21	0.78	0.39
Right posterior vidian canal (PVC)	0.62	1.01	0.44	1.03	0.78	0.33
Left hypoglossal canal (HC)	0.37	0.39	0.85	0.20	0.25	0.59
Right hypoglossal canal (HC)	0.51	0.95	1.00	0.15	0.66	0.67
Left ear canal lateral opening superior (ECLOS)	1.88	0.62	1.28	1.55	0.43	1.03
Left ear canal lateral opening inferior (ECLOI)	1.88	1.89	1.50	1.55	1.45	1.33
Left ear canal lateral opening anterior (ECLOA)	1.88	1.41	1.47	1.55	0.95	0.85
Left ear canal lateral opening posterior (ECLOP)	1.88	0.98	2.02	1.55	0.82	1.56
Left ear canal medial opening superior (ECMOS)	1.15	0.54	0.73	1.53	0.58	0.50
Left ear canal medial opening inferior (ECMOI)	1.15	0.66	0.79	1.53	0.62	0.56
Left ear canal medial opening anterior (ECMOA)	1.15	0.65	0.63	1.53	0.54	0.49
Left ear canal medial opening posterior (ECMOP)	1.15	0.66	0.75	1.53	0.63	0.43
Right ear canal lateral opening superior (ECLOS)	1.18	0.47	1.40	0.70	0.18	0.74
Right ear canal lateral opening inferior (ECLOI)	1.18	1.48	1.06	0.70	1.04	0.70
Right ear canal lateral opening anterior (ECLOA)	1.18	1.13	1.49	0.70	0.63	0.53
Right ear canal lateral opening posterior (ECLOP)	1.18	0.59	1.77	0.70	0.38	1.06
Right ear canal medial opening superior (ECMOS)	0.80	0.41	0.67	1.40	0.21	0.33
Right ear canal medial opening inferior (ECMOI)	0.80	0.49	0.49	1.40	0.24	0.35
Right ear canal medial opening anterior (ECMOA)	0.80	0.43	0.59	1.40	0.24	0.32
Right ear canal medial opening posterior (ECMOP)	0.80	0.49	0.59	1.40	0.39	0.35

**Table 2:** Change in distances between T1 and T2.

Distances	Change in distance (T2-T1)	
	Mean	SD
Left ECLOS to left ECMOS	−1.80	8.87
Right ECLOS to right ECMOS	−1.81	11.81
Left ECLOI to left ECMOI	0.21	2.94
Right ECLOI to right ECMOI	0.18	3.25
Left FO to left ECLOS	−2.97	12.38
Right FO to right ECLOS	0.73	10.04
Left FO to left ECMOS	−4.37	18.30
Right FO to right ECMOS	−3.39	17.39
Left FR to left ECLOS	0.51	4.61
Right FR to right ECLOS	1.30	5.04
Left FR to left ECMOS	−2.54	12.24
Right FR to right ECMOS	−2.12	11.69
Left HC to left ECLOS	0.53	2.95
Right HC to right ECLOS	1.14	3.85
Left HC to left ECMOS	−1.66	9.82
Right HC to right ECMOS	−1.04	8.73
FM to left ECLOS	0.71	3.29
FM to right ECLOS	0.43	2.87
FM to left ECMOS	−1.48	9.05
FM to right ECMOS	−0.48	3.85
Left ECLOS to left ECLOI	−1.35	12.38
Right ECLOS to right ECLOI	−2.37	14.80
Left ECLOP to left ECLOA	0.27	0.94
Right ECLOP to right ECLOA	0.30	0.99
Left ECMOS to left ECMOI	−1.22	5.22
Right ECMOS to right ECMOI	−1.23	5.19
Left ECMOP to left ECMOA	−0.31	0.63
Right ECMOP to right ECMOA	−0.22	0.71

**Table 3:** Patient demographics.

	Age at T1 (years)		Age at T2 (years)	
	Mean	SD	Mean	SD
Males (23)	13.6	1.4	16.7	1.4
Females (33)	12.7	1.2	15.5	1.3
Total (56)	13.1	1.5	16	1.3

## Data Availability

The data that support the findings of this study are available from the University of Alberta, Orthodontic Program, but restrictions apply to the availability of these data, which were used under license for the current study, and so are not publicly available. Data are however available from the authors upon reasonable request and with permission of the editor.

## Abbreviations

CBCT: Cone-beam computed tomography
2D: Two-dimensional
3D: Three-dimensional.
